# The ethical impact of Spock receiving ABA therapy

**DOI:** 10.3389/fpubh.2026.1777853

**Published:** 2026-03-25

**Authors:** Brittany Faith Thacker

**Affiliations:** Independent Researcher, Pikeville, KY, United States

**Keywords:** applied behavior analysis (ABA), ASD, ethical treatment, individualized treatment, level of support

## Introduction

This work is a clinically informed opinion piece and based on the experience and academic review of the author. This piece is intended to review ethical considerations and stigmatization on a treatment modality for autism spectrum disorder (ASD). Discussed information utilizes dimensions of applied behaviors analysis (ABA) framework for clinical practice presented by Baer et al. (1).

ABA therapy is the leading evidence-based treatment for ASD. While much scientific research has been done to signify the use of ABA, there remains a negative stigma in the social acceptance of ABA therapy at large. There are many possibilities for this widespread opinion and those that combat the efficacy of this medical service range from those who have simply heard about this form of treatment to those who have directly received the services. ABA also receives criticisms by many for the repetitive nature and the principle of conformity to rigid skill acquisition goals (2). Du et al. (3) suggests further research is needed to understand the potential impact on social and emotional domains of children's development who attend ABA therapy services. Leaf et al. (4) concludes that across methodologies improvements in treatment outcomes from the utilization of the science of behavior analysis has occurred.

Alternative or co-occurring therapies that have also shown validity for positive treatment outcomes for individuals diagnosed with ASD include Speech therapy. Due to impacts on communication being a diagnostic criterion for ASD, speech therapy plays a key role in treatment options as well. A recent review revelated that interventions for ASD delivered by Speech Language Pathologists have increased over the last decade (5).

One of the biggest components of ABA therapy is the individualized treatment approach. As all people diagnosed with ASD present with different topographies, the treatment must reflect the needs of the individual. The techniques and scientific approach those trained in ABA delivers can be applied to multiple types of behavior modification. The uniformity of strict rules of the science delivered in an individually functional way promotes the success of those receiving care.

Spock is a pop culture character created by Gene Roddenberry and first appeared in 1966 (6). While the diagnosis of ASD did not appear in the *DSM* until 1968, it is a current popular belief by fans of the Star Trek universe that this half-human Vulcan fits the criteria for an ASD diagnosis (7). Working under the assumption this diagnosis could be appropriately confirmed; Spock would not necessarily benefit from an ABA treatment approach.

As mentioned earlier, ABA is an individualized approach that works toward gaining milestones across language, social, and adaptive living domains while also eliminating barriers to learning including the systematic reduction of maladaptive behaviors. During the process of developing initial evaluation and treatment design for a client to begin receiving ABA therapy planning ahead is a vital part of the service plan. Having a planned transition of services to ensure generalization of learned skills across all settings is necessary for the successful utilization of ABA services.

With this in mind, it is also necessary to have adequate criteria for the appropriate discharge from services. One specific criterion is that the client has reached mastery of treatment goals (8). When considering intensive ABA therapy (typically at least 20 h per week for early intervention) goals would be set based on the needs and severity of need within each domain. Peterson et al. (9) conducted a study that investigates the relationship between participant age and number of hours in treatment weekly that produces most effective outcomes, further emphasizing the importance of individualized coordinated care planning.

When considering the character of Spock, they would be entering ABA services already meeting and most likely exceeding ethical standards for discharge criteria from intensive ABA therapy. While in general everyone can benefit from the scientific principle and strategies of ABA, the delivery of intensive methods to individuals who already fall into the discharge criteria for services would be unethical. There is also the risk that an unintentional element of indoctrination could develop with continued unnecessary practice of ABA therapy delivered to individuals who do not qualify for the service.

ASD is a lifelong diagnosis. While characteristics of this developmental disorder can be akin to the personality of an individual, the features of ASD vary widely creating the spectrum style diagnosis. There are three levels related to this diagnosis coordinating with perceived level of support to safely complete daily living tasks required by the individual. Level one suggests less support is required with the adult individual expected to live independently, level two requires daily support intermittently such as living in an individual apartment of an assisted living group home, and level three suggests substantial support and care is needed daily with no expectation of independently completing daily living tasks on a regular basis (10).

There have been limited studies on which diagnosed level can benefit the most from intensive ABA therapy. While misconceptions can easily deduce those with the most substantial needs benefit the most, it can also become an error in logic since all people can make gains from behavioral support. The most noticeable gains would occur in those requiring less support to achieve more functional independence. It is therefore more efficacious to presume Spock as the forerunner of beginning ethical practice standards of ABA (a science driven, evidence-based treatment) to the Starship Enterprise rather than being the receiver of the therapy.

To better achieve positive outcomes, responsibility falls on the providers to adequately evaluate their own methods of assessment for appropriateness of ABA services. Teaching a client the methods in which they learn most efficiently and then taking a step back to evaluate if ABA is holding them back after hitting this benchmark. The rigidity in the teaching methods and opportunities for regression due to peers modeling maladaptive behavior in the clinic setting can create adverse effects for individual clients getting close to or exceeding the discharge criteria for services. Self-evaluation of methods and outlook for the personal place in the field is necessary for providers at regular intervals due to the position of instructional control and reinforced compliance built with clients. This evidence-based treatment is most effective when practiced by individuals in a competent and respectful way to not do a disservice to an already vulnerable population.

ABA is not a one size that fits all brand of therapy, and the unique beauty is that this treatment is specifically tailored to each client. There is a possibility that if a client receives effective ABA therapy and reports a negative experience then the client in question was not a good fit as a candidate for this intensive treatment method. Providing effective services to those who are a good fit, as well as understanding the signs of someone with a diagnosis not meeting the ethical criteria can allow the field of ABA to better adhere to the Vulcan salute and wish all to, “Live long and prosper” (6).

## Limitations

The case study presented is entirely metaphorical by nature and is intended to assist in concept interpretation and the understanding of the ethical dynamics within a treatment methodology. While this character is known in popular culture and has had a resurgence of influence for multi-generational understanding, limitations including speculative diagnostics analysis should be acknowledged.
